# Supplementary material for: Collagen Lattice Model, Populated with Heterogeneous Cancer-Associated Fibroblasts, Facilitates Advanced Reconstruction of Pancreatic Cancer Microenvironment
Source: Int J Mol Sci. 2024 Mar 27;25(7):3740. doi: 10.3390/ijms25073740 (PMC11011612; doi:10.3390/ijms25073740)
Supplement: Supplementary file 1 [file ijms-25-03740-s001.zip › ijms-2906448-supplementary.docx]

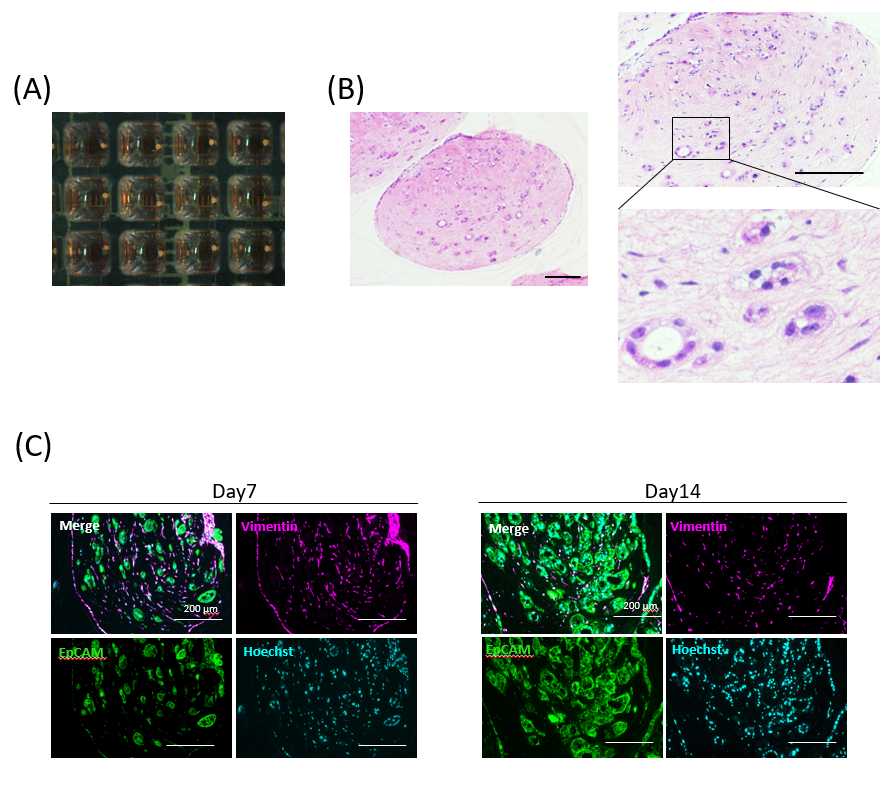
**Supporting Information**

**Supplementary Figure S1**. The construction of comPDAC-FPCL model in 384-well plates. (A) Gel contraction in the comPDAC-FPCL model is present in 384-well plates. (B) Hematoxylin and eosin staining of comPDAC-FPCL models on Day 7 in 384-well plates. Scale bar: 200 μm. (C) Immunofluorescence staining of comPDAC-FPCL models (Days 7 and 14). Capan-1 cells stained for EpCAM (Green), CAF progenitors stained for vimentin (Magenta), and nuclear stain for Hoechst (Cyan). Scale bar: 200 μm.


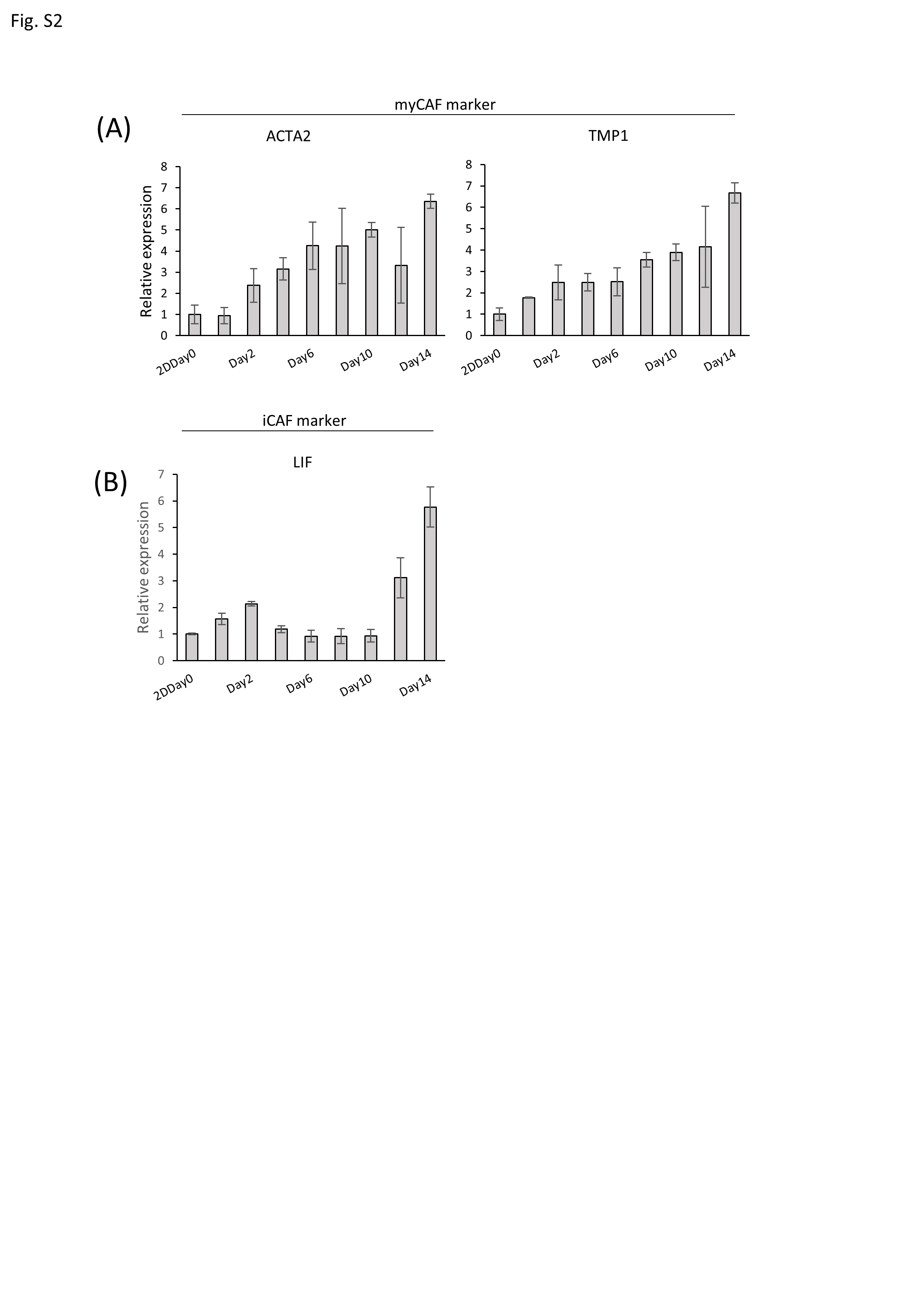
**Supplementary Figure S2.** Dynamic changes in gene expression during time-course experiments. (A) Dynamic changes in myCAF-related gene expression during time-course experiments. Results are presented as mean ± standard deviation (SD; n=3). (B) Dynamic changes in iCAF-related gene expression during time-course experiments. Results are presented as mean ± SD (n=3).

**Supplementary Table S1.** Gene Ontology of increased biological processes in comPDAC-FPCL models on Day 7 (Day 7 vs Day 0)


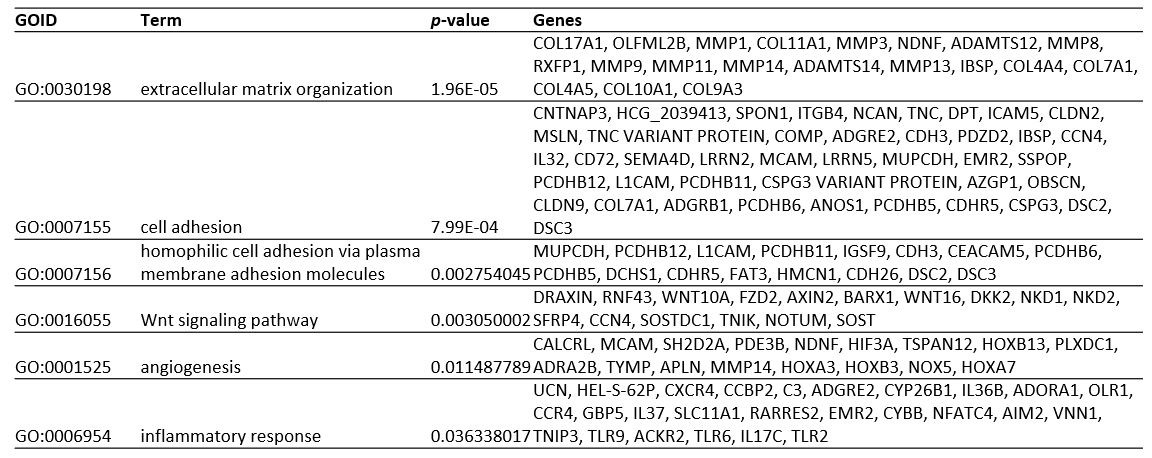


**Supplementary Table S2.** Gene Ontology of increased biological processes in comPDAC-FPCL models on Day 14 (Day 14 vs Day 0)


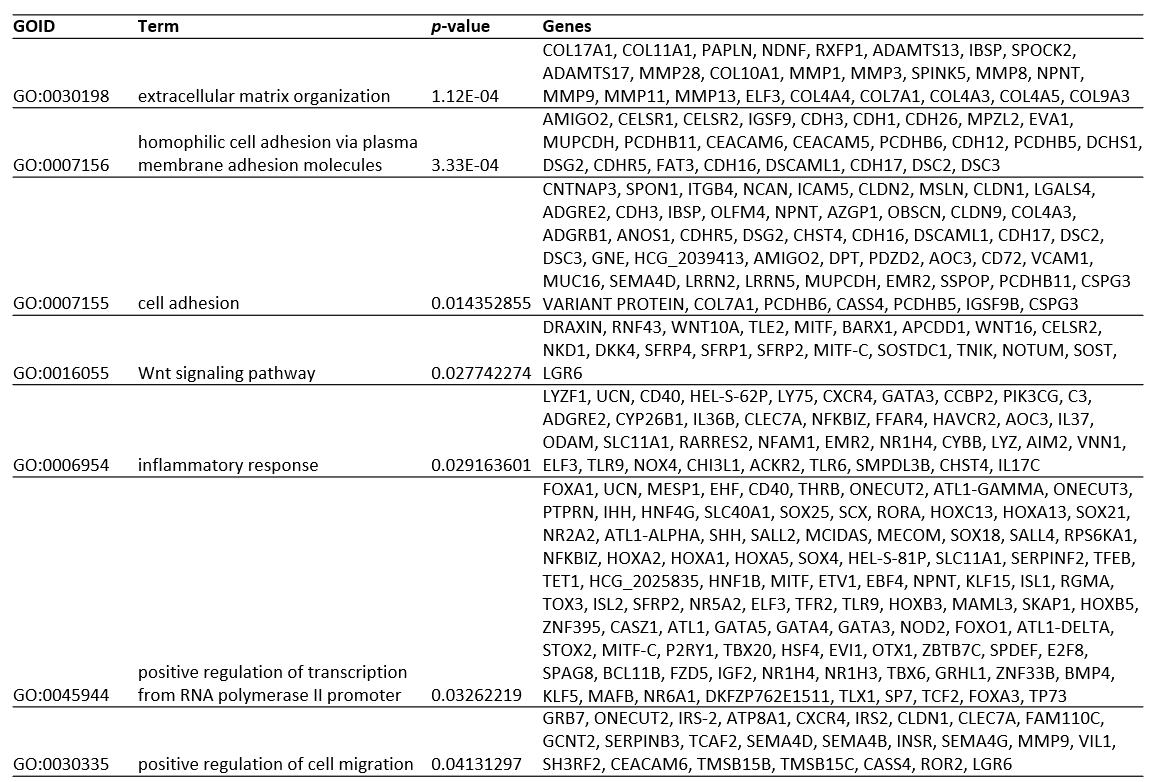


**Supplementary Table S3.** Gene Ontology of increased biological processes in comPDAC-FPCL models on Day 7 (Day 7 vs Day 14)


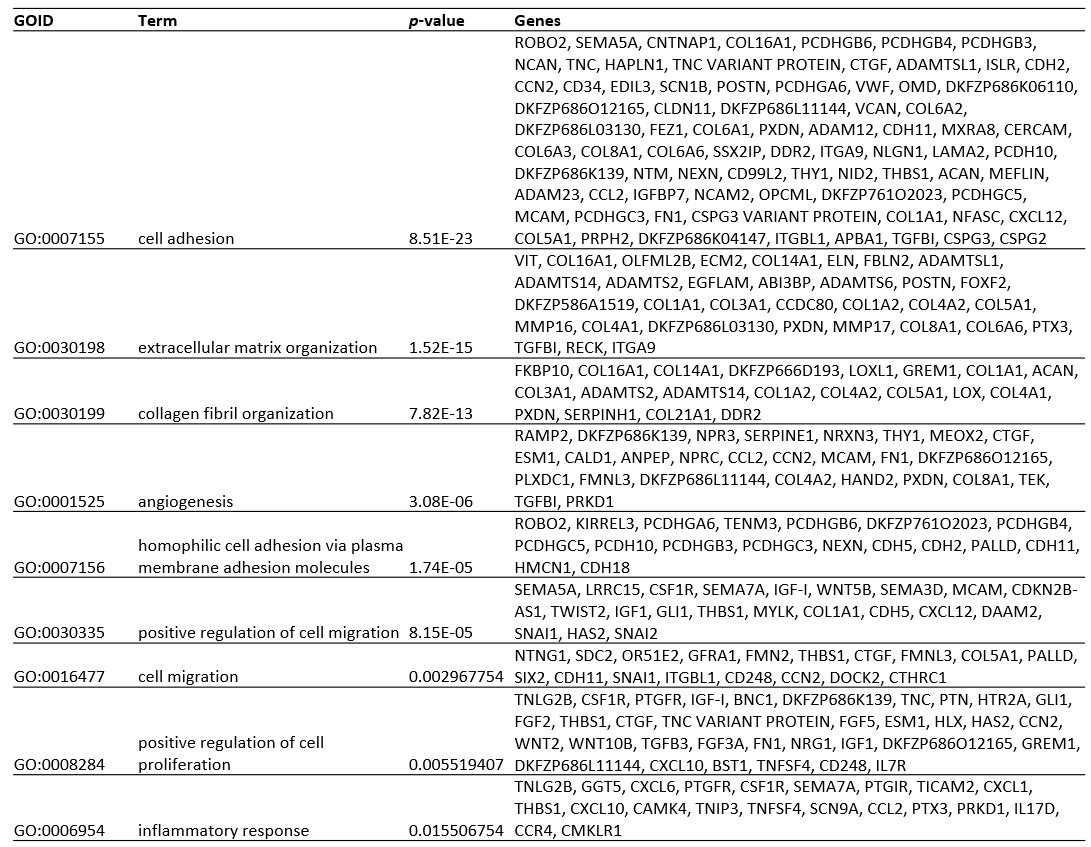


**Supplementary Table S4.** Gene Ontology of increased biological processes in comPDAC-FPCL models on Day 14 (Day 7 vs Day 14)


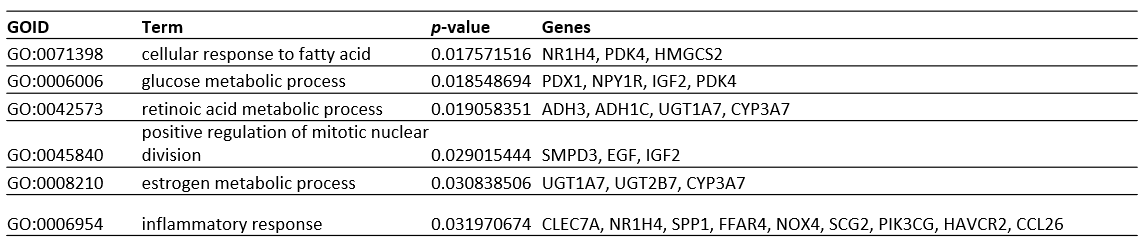


**Supplementary Table S5.**
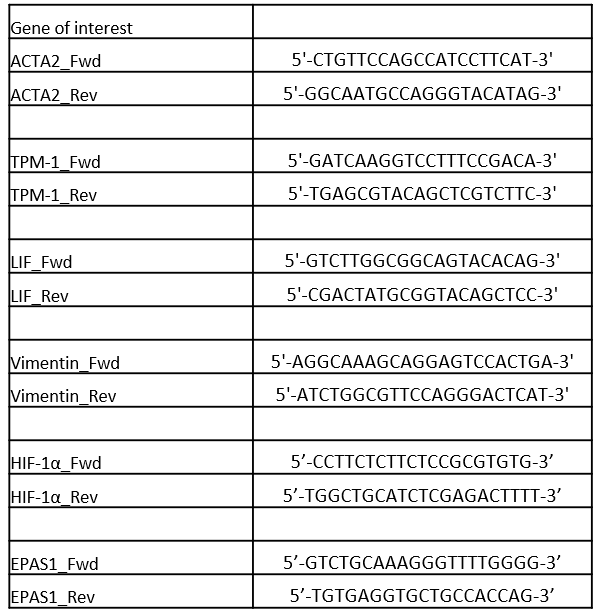
The primer sequences for qPCR analysis
